# Supplementary material for: Assessment of miRNAs as transcriptional regulators in respiratory syncytial virus infection through computational analysis and molecular docking studies
Source: PLoS One. 2026 Mar 30;21(3):e0345571. doi: 10.1371/journal.pone.0345571 (PMC13035128; doi:10.1371/journal.pone.0345571)
Supplement: S1 File — (DOCX) [file pone.0345571.s002.docx]

**Supplementary Materials**

**Assessment of miRNAs as Transcriptional Regulators in Respiratory Syncytial Virus Infection through Bioinformatics Analysis and Molecular Docking Studies**

Mubashir Hassan^1^, Muhammad Shahzad Iqbal^2^, Muhammad Yasir^3^, Wanjoo Chun^3^, Zainab Yaseen^4^, Saba Shahzadi^1^, Mark E Peeples^5^ and Andrzej Kloczkowski^1,6,7^*

^1^The Steve and Cindy Rasmussen Institute for Genomic Medicine at Nationwide Children’s Hospital, Columbus, OH 43205, USA.

^2^Department of Biochemistry, University of Okara, Okara 56300, Pakistan

^3^Department of Pharmacology, Kangwon National University School of Medicine, Chuncheon 24341, Republic of Korea

^4^Department of Biotechnology, Faculty of Science and Technology (FOST), University of Central Punjab, Johar Town, Lahore, Pakistan

^5^Center for Vaccines and Immunity, Abigail Wexner Research Institute, 700 Children's Drive, Columbus, OH 43205

^6^Department of Pediatrics, The Ohio State University Columbus, OH 43205, USA

^7^Department of Biomedical Informatics, The Ohio State University, Columbus, OH, 43210, USA

**Corresponding Author:**

**Andrzej Kloczkowski**:

Email: [Kloczkowski.1@osu.edu](mailto:Kloczkowski.1@osu.edu)


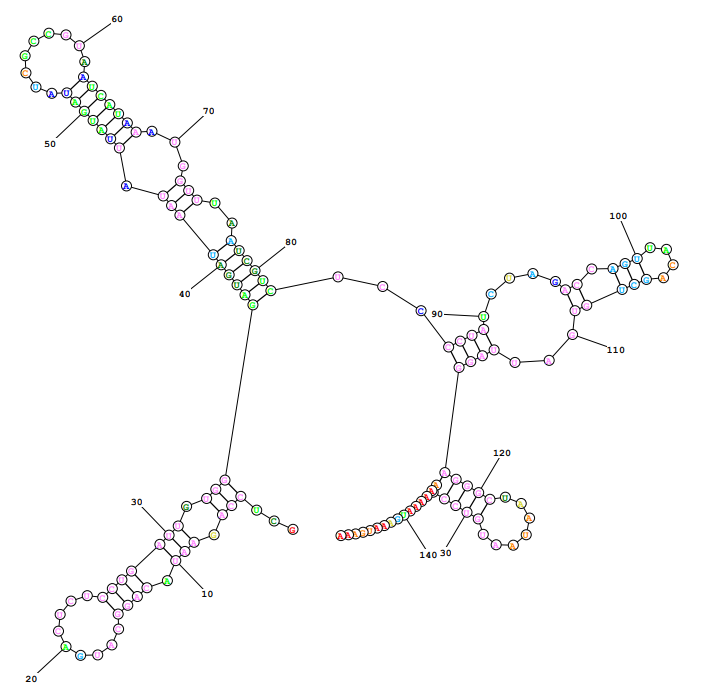


**Figure S1**. The predicted secondary structure of mRNA
