# Supplementary figures and images for: Assessment of miRNAs as transcriptional regulators in respiratory syncytial virus infection through computational analysis and molecular docking studies
Source: PLoS One. 2026 Mar 30;21(3):e0345571. doi: 10.1371/journal.pone.0345571 (PMC13035128; doi:10.1371/journal.pone.0345571)

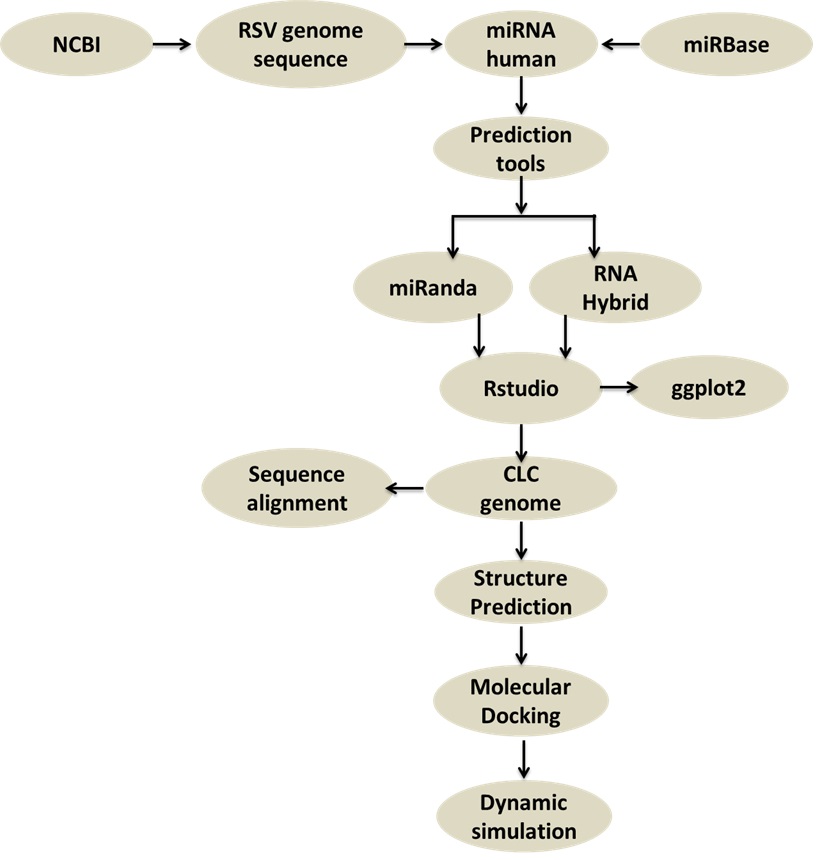

Supplement: S1 Fig — (JPG) [file pone.0345571.s003.jpg]
